# Infrared-based visualization of exhalation flows while wearing protective face masks

**DOI:** 10.1063/5.0076230

**Published:** 2022-01-25

**Authors:** E. Koroteeva, A. Shagiyanova

**Affiliations:** Faculty of Physics, Lomonosov Moscow State University, Moscow 119991, Russia

## Abstract

Since the onset of the COVID-19 pandemic, a large number of flow visualization procedures have been proposed to assess the effect of personal protective equipment on respiratory flows. This study suggests infrared thermography as a beneficial visualization technique because it is completely noninvasive and safe and, thus, can be used on live individuals rather than mannequins or lung simulators. Here, we examine the effect of wearing either of three popular face coverings (a surgical mask, a cloth mask, or an N95 respirator with an exhalation valve) on thermal signatures of exhaled airflows near a human face while coughing, talking, or breathing. The flow visualization using a mid-wave infrared camera captures the dynamics of thermal inhomogeneities induced by increased concentrations of carbon dioxide in the exhaled air. Thermal images demonstrate that both surgical and cloth face masks allow air leakage through the edges and the fabric itself, but they decrease the initial forward velocity of a cough jet by a factor of four. The N95 respirator, on the other hand, reduces the infrared emission of carbon dioxide near the person's face almost completely. This confirms that the N95-type mask may indeed lead to excessive inhalation of carbon dioxide as suggested by some recent studies.

Respirable aerosolized particles and droplets, from 0.05 to 500 *μ*m in diameter, which infected individuals generate during talking, breathing, coughing, and sneezing,^1^ are acknowledged as the primary source of transmission of various bacterial and viral infections, including the new coronavirus COVID-19.^2–4^ To reduce the risk of cross-infection via droplet dispersion, the use of protective face coverings is widely recommended by public health officials ever since the emergence of the ongoing COVID-19 pandemic.^5^ All the known types of coverings act as physical barriers for respiratory droplets that leave the wearer's nose and mouth. None of them, however, offer 100% protection, and the smaller the size of the virus-laden fluid particles, the better they can penetrate the mask material.^6,7^

Most of the current knowledge on the effect of face coverings on respiratory droplet dynamics is based on numerical^8–11^ and experimental flow visualization.^12–16^ As the majority of the quantitative flow visualization techniques are intrusive and may be unsafe for live human volunteers [i.e., particle image velocimetry (PIV) tracers or “smoke” particles may be irritating and toxic to inhale], the cited experimental studies mainly use mannequins or human lung simulators rather than live individuals. The extrapolation of simulated respiratory flows to real-life scenarios, however, is not straightforward. The noninvasive visualization of exhaled airflow may be performed using shadow or schlieren techniques.^12,17,18^ These techniques detect changes in the refractive index (flow density) induced by warm air exiting the lungs during an exhalation event, but, unfortunately, they cannot distinguish between exact sources of these changes.

Infrared thermography is another completely noninvasive and safe visualization technique that, unlike schlieren imaging,^17^ does not require even additional optical elements. This technique allows obtaining real-time two-dimensional temperature distributions by measuring the thermal radiation emitted from objects. Biomedical application of infrared thermography so far mostly involved visualization of thermal fields on the human skin (i.e., surface temperatures).^19,20^ With the outbreak of the COVID-19 pandemic, the use of mobile infrared devices is showing an exponential growth; however, their sensitivity and reliability in mass screening of fever remain a key concern.^21^

On the other hand, the ability of infrared cameras to visualize gas flows under certain conditions leads to another potential approach to exploit infrared imaging. In particular, due to the carbon dioxide (CO_2_) absorption band centered around 4.3 *μ*m, any changes in its concentration are detectable by any thermal camera operating within a mid-wave (∼3–5 *μ*m) infrared range. This provides a means for effective tracking of human respiratory activity based on infrared thermography^22^ (see the Appendix). In this study, we use the infrared-based approach to visualize the flow exhaled during breathing, talking, and coughing by capturing the increased thermal emission of carbon dioxide near a person's face. To the best of our knowledge, it is the first time that time-resolved infrared thermography is employed to compare the dynamics of thermal inhomogeneity due to respiratory activities of a person while wearing a protective face mask.

The subjects were recruited from a group of seven healthy volunteers aged 27.6 ± 4.7 years. Prior to participation, all subjects gave their informed consent about the experimental procedure. Both authors also served as volunteers. The data acquisition protocol was approved by the Ethical commission of Lomonosov Moscow State University.

The experiments were conducted in an isolated laboratory room with no windows (no direct sunlight) and also no ventilation or external airflow during the acquisition. The measured room temperature was 22 ± 1 °C, with the relative humidity of 55 ± 3%. The environmental conditions did not change during the experiments. The participants were comfortably seated upright in front of a black screen (serving as a uniform thermal background), 80–90 cm from the thermal camera, either full-face or in profile to the camera. The participants were asked to maintain a relatively constant position, although a slight head movement was unavoidable (especially during coughing).

The thermal imaging was performed using a cooled FLIR SC7700 camera at frame rates of 15–50 Hz. The camera had a 640 × 512 focal plane array photon detector sensitive in the 3.7–4.8 *μ*m (mid-wave infrared) spectral range. Depending on the needed field-of-view, the camera was equipped with a 25 or a 50 mm lens. For the data post-processing, we used the FLIR ResearchIR Max software package. The infrared camera was employed for detecting thermal variations induced by the presence of an exhaled respiratory jet rather than temperature measurement. Thus, the accuracy of the present method relied on the camera's thermal sensitivity—the Noise Equivalent Temperature Difference (NETD) parameter (< 0.025 K at room temperature).

The acquisition procedure was as follows. At first, we recorded the thermal videos of the subject without wearing a mask. The subject was invited to breathe normally through the nose for up to 2 min, then to talk for 1 min, and then perform forced coughing for another 1 min. We kept 3–5 min intervals between each phase. After that, the same procedure was repeated while the subject was wearing, one after another, a surgical (SM), a cloth (CM), and an N95 mask that yielded four series of experiments for each subject. The face masks were put on correctly and always covered the subject's nose and mouth.

The first set of measurements was conducted to test the assumption that wearing a face mask can affect the respiratory rate. We recorded and post-processed both full face and profile thermal images of subjects breathing normally through the nose while wearing three types of face masks and compared the results with the baseline data (without a mask).

As shown earlier,^22,23^ the respiratory characteristics can be tracked using mid-wave infrared cameras based on two physical mechanisms. The first mechanism is the increase in skin temperature near the nostrils due to the periodic exhalation of warm air from the lungs. This mechanism is predominantly used in the infrared-based methods of tracking human respiratory activity.^24–26^ The second mechanism is based on the increased concentration of carbon dioxide in the exhaled air (about a factor of 100 compared to typical concentrations in the surrounding fresh air).

Figures 1 and 2 show sample plots of a mean thermal signal at given regions of interest (ROIs) together with respective thermal images. For better readability, each plot is presented as a variation from the average value of apparent temperature. As expected, a protective mask acts as a breathing sorption indicator,^27^ sufficiently enhancing the signal amplitude and increasing the size of ROIs from which useful thermographic data can be collected. As masks are typically made of material with low specific heat, they rapidly respond to changes in thermal flux and correctly reflect the breathing activity. Thus, the periodical variation in thermal signal during normal breathing can be captured without the correction of subtle head or face muscle movements that is usually required in infrared-based breathing diagnostics.^26^

**FIG. 1.**
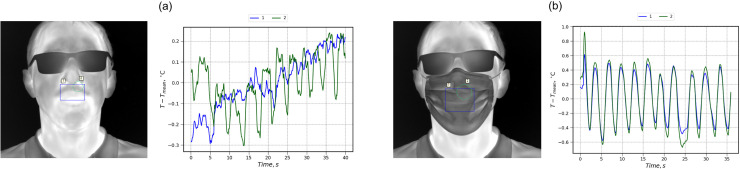
Changes in ROIs temperature with time during breathing without the correction for head movement (full face): (a) no mask; and (b) surgical mask.

**FIG. 2.**
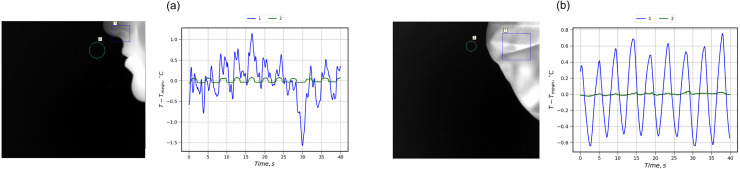
Changes in ROIs temperature with time during breathing without the correction for head movement (profile): (a) no mask; and (b) cloth mask.

In profile thermal images (Fig. 2), we selected several ROIs on the background close to the face. The mean thermal signal from these ROIs over time yielded low-amplitude curves that follow the higher amplitude signal from the mask surface. Both surgical and cloth masks allowed the flow with increased concentration of carbon dioxide to pass through the mask resulting in a temperature inhomogeneity captured by the thermal camera even during normal breathing.

The breathing rates were calculated from the extracted waveforms of the thermal signal using the Fast Fourier Transform algorithm. The results in the actual value of the breathing rate varied between individuals, from 12.0 to 23.6 breaths per min. Both slight increase and decrease in breathing rates compared to the baseline, as well as no change, were recorded. No statistical difference between a surgical, a cloth, or an NP95 mask was observed. We calculated the value *dR* (breaths per min) that indicated the change in a breathing rate with a mask (*R_m_*) compared to the normal breathing rate without a mask (*R*_0_) for each participant, $dR=R_{0}-R_{m}$. Across all the experiments, the value of *dR* was 0.34 (breaths per min) with the standard deviation of 2.46 (breaths per min), which basically implies that wearing a protective mask does not affect the breathing rate in healthy people.

The second set of experiments was conducted to study the dynamics and spreading of exhaled flows when wearing a protective mask. From the fluid dynamics point of view, nasal breathing produces two conical jets, approximately 1 cm in diameter, while coughing, sneezing, and talking produce a single jet-like flow, around 2 cm in diameter.^28^ In air near a human face, the further evolution of an exhaled jet is dominated by forced and then natural convection.

The thermal imaging revealed that, compared to breathing, speaking, or coughing without a mask, both cloth and surgical masks restrict the forward outflow of the exhaled air, and the flow of carbon dioxide near a face is visualized propagating vertically above the mask, near the person's nose, and eyes (Fig. 3). For all thermal images, the contrast was enhanced by extracting the first frame (before exhalation) from the image sequences. Clearly, neither a surgical nor a cloth mask can provide a complete airtight fit, and a part of exhaled air (containing carbon dioxide) escapes through the small gaps between a mask and a face.

**FIG. 3.**
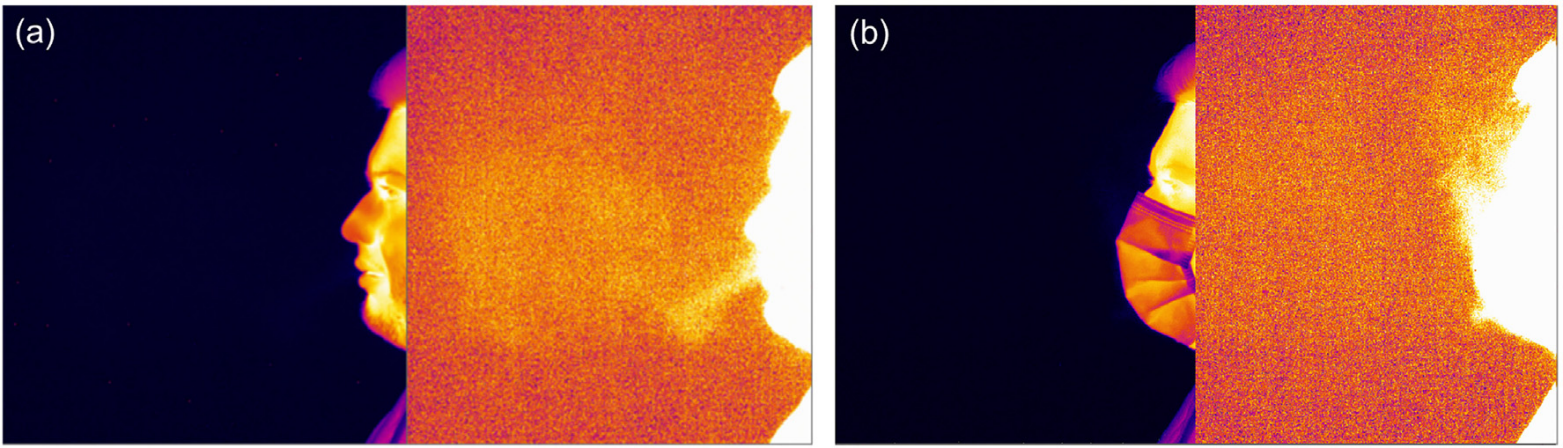
Infrared image of a single coughing act (a) without a mask (multimedia view) and (b) while wearing a surgical mask. The right-side images are obtained by background subtraction and contrast enhancement. Multimedia views: https://doi.org/10.1063/5.0076230.1
10.1063/5.0076230.1; https://doi.org/10.1063/5.0076230.2
10.1063/5.0076230.2

As for the N95 respirator (Fig. 4), almost no thermal inhomogeneity outside the mask could be captured during breathing and coughing. Figure 4(b) shows variations in the apparent temperature of the ROI located near the mask for the N95 respirator compared to coughing in surgical (SM) or cloth (CM) mask. Although the amplitudes of recorded thermal variations were only a fraction of a degree, for the case of breathing or coughing in either surgical or cloth masks they were at least 10 times higher than the noise level. As for the N95 mask, the temperature variations near the mask were comparable to the noise level and the camera's NETD value. The thermal imaging suggests that an N95 mask with an exhalation valve partially traps carbon dioxide preventing its free outflow from the face. This result is in agreement with some current studies showing increased CO_2_ levels inside an N95-type respirator.^29,30^

**FIG. 4.**
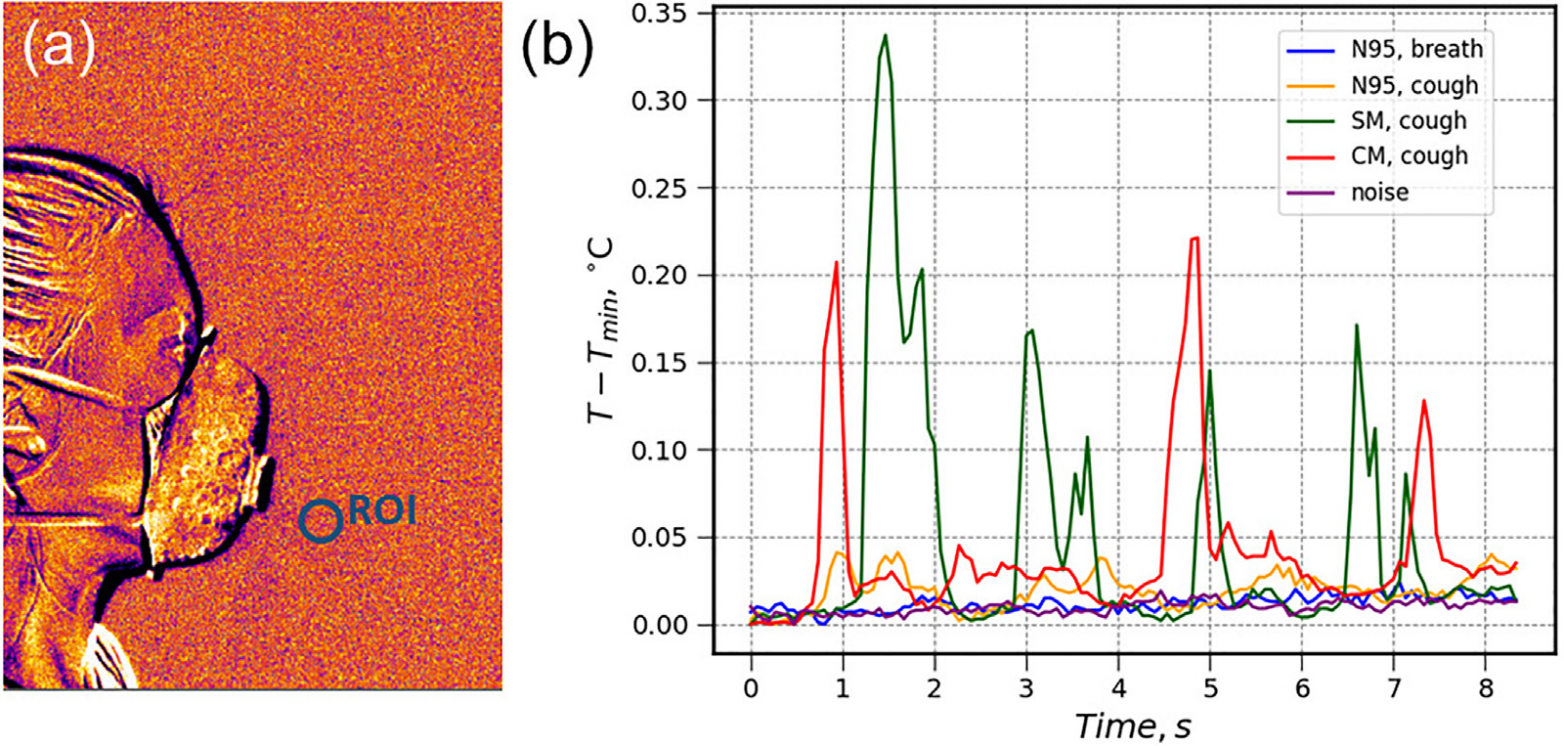
(a) Thermal image of a person breathing with an N95 mask; and (b) variations in the ROI signal for breathing and coughing with an N95 mask (N95), coughing with a surgical mask (SM), and coughing with a cloth mask (CM). Typical background noise is shown for comparison.

To provide a more quantitative comparison between the no mask and mask scenario, we post processed the thermal sequences of coughing process recorded at a rate of 50 frames/s to extract the initial exit velocity of the cough jet. For that, we tracked the propagation of thermal inhomogeneity (level threshold) in the first few frames of a single coughing act (Fig. 5). We need to point out that small variations in CO_2_ concentrations during exhalation processes are hard to detect in still images. However, we used the fact that the image processing within the human brain is more sensitive to changes in its field of view so the propagation of CO_2_ concentrations was better tracked on time-resolved sequences. Still, the uncertainty in detecting the jet edge was relatively high and reached 10–20 pixels on some images. The variations of the jet exit velocity from different coughing acts of the same participant and between the participants were of the same order. Thus, the presented values are the average among different coughing acts for all the participants. The overall uncertainty was estimated as the combination of image processing uncertainty and the standard deviation.

**FIG. 5.**
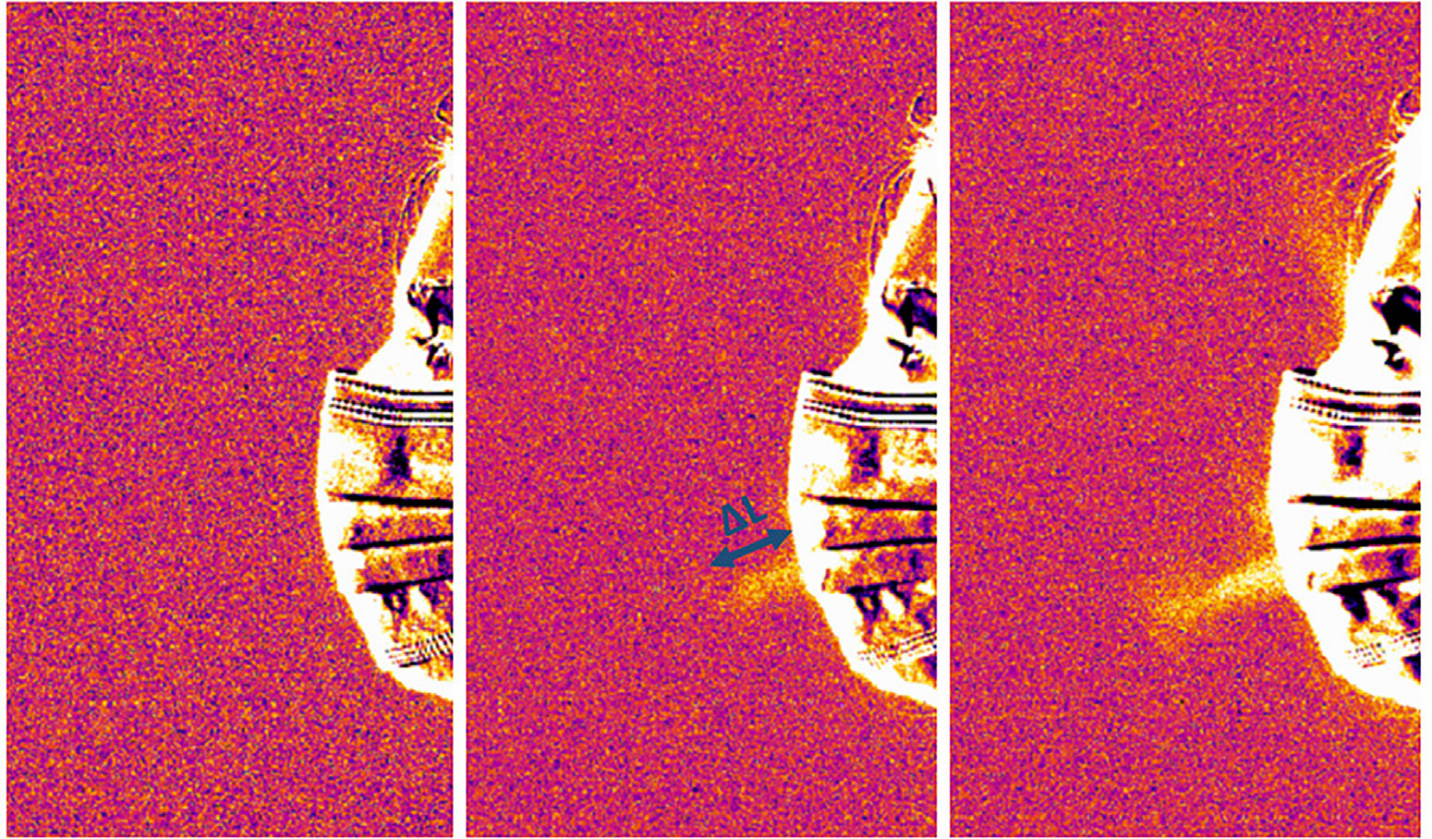
Three sequential frames capturing the onset of a single coughing act. Time between frames—0.02 s.

As the coughing velocity is known to be highly variable under different conditions and from subject to subject,^31^ of interest was the change in the initial cough velocity due to wearing a mask rather than its absolute values. For the case with no mask, the average initial exit velocity of the exhaled jet was 3.18 ± 1.01 m/s. This value is consistent with the results of earlier PIV^31^ and shadow imaging,^32^ although we did not observe any statistically significant difference between male and female subjects. The measured velocity of a jet exhaled through a medical mask was about four times smaller (0.79 ± 0.24 m/s). We also performed a few measurements using medical masks worn for 2–3 h and observed a slight (less than 9%) increase in the exit velocity compared to a new mask (0.86 ± 0.20 m/s). Compared to medical masks, locating a single exhaled jet was challenging with cloth masks as the visualized outflow highly depended on the mask design and fabric. (Participants were asked to bring their own cloth masks.) For example, some masks that left larger gaps with the face allowed the exhaled jet to escape faster in the upward direction while some masks led to “spreading” of the jet that passed through the fabric.

As a final remark, we need to stress that the present infrared-based method detects only increased carbon dioxide concentrations in air produced during exhalation process (see the Appendix for more detail). Although the aerosols and droplets can also emit or scatter infrared radiation, a thermal camera itself cannot distinguish between sources of radiation (gases, aerosols, or droplets). Since the sizes of airborne particles are orders of magnitude larger than air molecules (such as CO_2_), their dynamics does not entirely follow the dynamics of exhaled air. However, infrared thermography still presents a viable tool that can provide additional insight into the physical properties of personal virus-protective equipment.

The presented results can be summarized as follows:

1. The presence of any protective mask on a face facilitates the use of infrared-based techniques for remote monitoring of breathing rate and breathing rate variability.
2. No evidence has been found that wearing a face mask by itself affects the breathing pattern in healthy humans.
3. The presence of a protective mask sufficiently affects the spreading of the respiratory jet flows. Both cloth and surgical masks restrict forced convection, and most of the exhaled air escapes in the upward direction through the holes at the edges of a mask. The initial velocity of a cough jet in the forward direction is reduced by about a factor of four.
4. Wearing N95 respirators, on the other hand, demonstrates near-zero concentrations of carbon dioxide around the subject's nose and mouse during coughing and breathing. This confirms that the N95-type mask may indeed prevent the free outflow of carbon dioxide through the mask.

## Figures and Tables

**FIG. 6.**
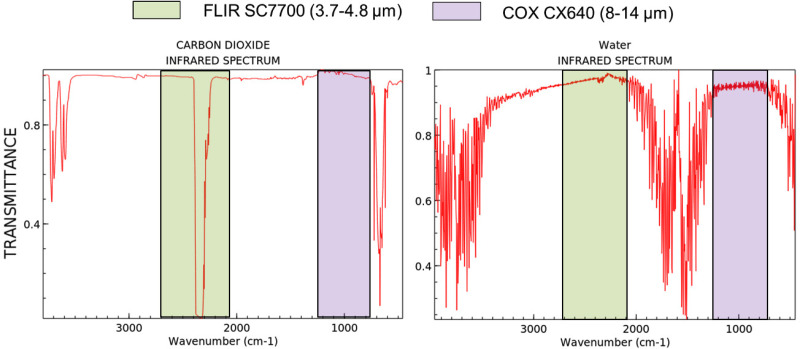
Carbon dioxide and water vapor infrared transmittance spectra (taken from the NIST Chemistry Webbook https://webbook.nist.gov/chemistry). The working bands of the mid-wave (FLIR) and the long-wave (COX) infrared detectors are highlighted.

**FIG. 7.**
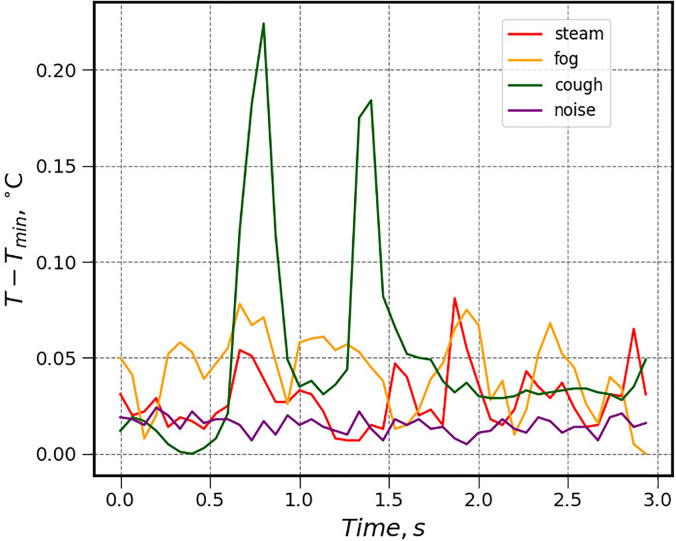
Thermal signal variations for a fog jet, a steam jet, and a cough jet recorded using the FLIR SC7700 camera. The recording frequency is 15 Hz.

**FIG. 8.**
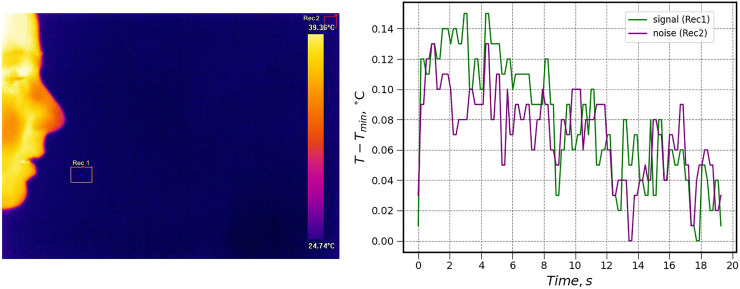
Thermal signal variations during several coughing acts (no mask) recorded using the COX CX640 camera (8–14 *μ*m).

## Data Availability

The data that support the findings of this study are available on request from the corresponding author.

## APPENDIX: CO_2_ VS H_2_O EMISSION

As the present study suggests, the changes in thermal signal arise from increased concentration of carbon dioxide in the exhaled air. This assumption, however, should be properly assessed, since water vapor is also infrared-absorptive and its content also changes during respiratory activities.

Figure 6 shows that the FLIR SC7700 camera used in this study should be much less sensitive to the presence of water vapor than carbon dioxide. To test this, we conducted separate experiments to compare the thermal imaging of water vapor to that of the exhaled air. The thermal camera (FLIR SC7700) was placed 80 cm in front of a uniform blackbody screen at room temperature. A vertical fog jet between the camera and the background was produced using an ultrasound fog generator (filled with water at 35 °C). To increase the possible contrast, we also performed thermal imaging of a horizontal water steam jet ejected from a distance of 1 m from the camera's field of view. Then, we asked a volunteer to perform a few coughing acts without a mask in front of the screen. The results comparing thermal signals with highest fluctuations recorded for a fog jet, a steam jet, and a cough jet are shown in Fig. 7. Although both the fog and the steam generators produced jets of water vapor visible to the naked eye, the recorded thermal fluctuations were several times less than those in the air exhaled by the human participant.

To further examine the emission source and the choice of the infrared detector, we have performed additional experiments using a COX CX640 infrared camera with an uncooled microbolometer detector. As shown Fig. 6, the camera's working range (8–14 *μ*m) is outside the absorption peak of carbon dioxide, but the absorption of water vapor in this range is slightly higher than in the 3.7–4.8 *μ*m range. The results of thermal imaging of several sequential coughing acts (without a mask) are given in Fig. 8. The thermal variations near the person's mouth during coughing cannot be distinguished when compared to the background signal. These results confirm that the water vapor infrared emission can be neglected in the present study. Moreover, the thermal imaging of human respiratory activities is expected to be most informative when using mid-wave infrared cameras preferably equipped with narrowband filters around the 4.3 *μ*m absorption band of CO_2_.
